# Technical Review on Endoscopic Treatment Devices for Management of Upper Gastrointestinal Postsurgical Leaks

**DOI:** 10.1155/2023/9712555

**Published:** 2023-06-12

**Authors:** Renato Medas, Eduardo Rodrigues-Pinto

**Affiliations:** ^1^Gastroenterology Department, Centro Hospitalar São João, Porto, Portugal; ^2^Faculty of Medicine of the University of Porto, Porto, Portugal

## Abstract

Upper gastrointestinal postsurgical leaks are challenging to manage and often require radiological, endoscopic, or surgical intervention. Nowadays, endoscopy is considered the first-line approach for their management, however, there is no definite consensus on the most appropriate therapeutic approach. There is a wide diversity of endoscopic options, from close-cover-divert approaches to active or passive internal drainage approaches. Theoretically, all these options can be used alone or with a multimodality approach, as each of them has different mechanisms of action. The approach to postsurgical leaks should always be tailored to each patient, taking into account the several variables that may influence the final outcome. In this review, we discuss the important developments in endoscopic devices for the treatment of postsurgical leaks. Our discussion specifically focuses on principles and mechanism of action, advantages and disadvantages of each technique, indications, clinical success, and adverse events. An algorithm for endoscopic approach is proposed.

## 1. Introduction

Upper gastrointestinal (UGI) postsurgical leaks (PSLs), defined as abnormal communications between the intraluminal and extraluminal compartments because of a defect in the integrity of the gastrointestinal wall, are devastating complications of surgery. Their occurrence negatively impacts postoperative outcomes, as they are the strongest independent risk factor for postoperative mortality [1]. They also delay oral feeding initiation, increase length of stay, risk of anastomotic stricture, and risk of re-operation up to 60% [2].

**Figure 1:**
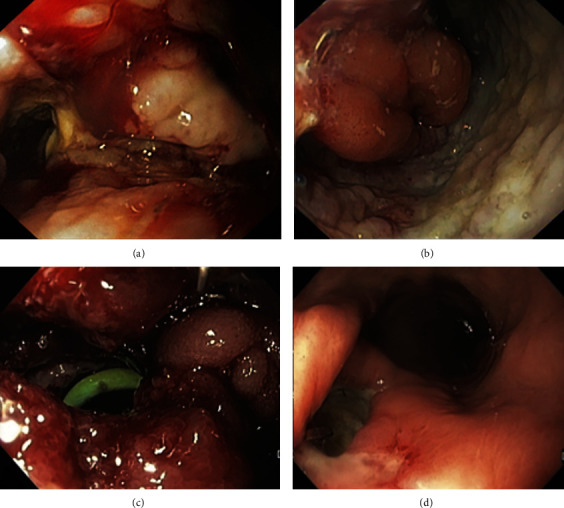
Endoscopic images of post-esophagectomy leak (a), post-gastrectomy leak (b), post-gastric bypass leak (c), and post-sleeve leak (d).

Frequency of UGI PSL is higher in cervical anastomosis than in intrathoracic anastomosis [2, 3], and in oncologic leaks than in bariatric leaks (esophagectomy: 8–26% [4], 3–12% after total gastrectomy: 3–12% [5]; Roux-en-Y gastric bypass (RYGB): 0.7–5%; sleeve gastrectomy (SG): 1–2% [6, 7]) (Figure 1). Leaks may occur immediately post-surgery or, more commonly, several weeks later. Acute leaks are usually related to technical issues, while delayed leaks often reflect healing insufficiencies due to ischemia at the staple-line or anastomosis [8–10].

PSLs are challenging to manage and often require radiological, endoscopic, or surgical intervention [11]. Their management should be based on several factors, with patient stability and time from surgery being probably the most important [11]. Historically, PSLs were managed either by rescue surgery, when the defect was present within the first 7–10 days, or a watch-and-wait strategy followed by secondary surgery if symptoms persisted. In stable patients, conservative and radiological interventions lead to highly variable rates of spontaneous closure, ranging from 16% to 46% [6, 12]. In patients who undergo rescue or redo surgery, mortality increases to 15–30%, with recurrence occurring in 13–33% of these patients with an added mortality of 9–30% [13]. Cost of care also has a 10-fold increase in these patients.

Nowadays, endoscopy is considered the first-line approach for the management of PSL [14, 15], as it seems to be associated with an improved outcome and better quality of life [16]. Recent studies have demonstrated the safety and efficacy of endoscopic interventions to manage transmural defects as first-line therapy (Table 1) instead of conventional modalities to either avert surgery or optimize patients for definitive future surgery [17]. However, there is no definite consensus on the most appropriate therapeutic approach in the management of PSL. Due to lack of an algorithmic endoscopic approach, these interventions are often applied in a stepwise manner or an institutional expertise–dependent manner [18].

The approach to a PSL should focus on clinical presentation, characteristics, and chronicity of the leak, correcting the underlying physiologic defect that predisposed and perpetuated the leak, minimizing the risk of chronic fistula formation, preserving the patient's ability to have enteral nutrition, and minimizing the use of costly, less effective endoscopic accessories and endoscopies.

Multiple endoscopic sessions are often required, and the strategy must be continually adapted based on the patient's anatomy, physiology, and response to therapy. The lack of defined criteria, such as size of the leak or existence of a wound cavity, poses a challenge for the choice of the best endoscopic treatment strategy.

Considering the multiplicity of endoscopic therapeutic options available and the need of tailoring each treatment, we aim to provide a technical review of the endoscopic devices available for the treatment of PSL, summarize the best options for each clinical situation, and propose an algorithm for endoscopic approach.

## 2. Leaks Specificities

Anatomic and physiologic factors, apart from technical errors, are responsible for the development of leaks. Intrinsic esophageal anatomy with the lack of an esophageal serosa and the negative pressure within the thoracic cavity may contribute to the development of post-esophagectomy leaks [19]. Sufficient blood supply [20] and adequate tension on the anastomosis site [20, 21] are essential for proper healing.

While foreign body material (staples, sutures, percutaneous drains) hampers proper healing, downstream obstruction distal to the surgical anastomosis, such as anastomotic strictures [22], narrowing at the incisura angularis or twisted/kinked stomach [22, 23] results in a higher pressure proximally, predisposing to a leak at the area of least resistance. Evidence about the effect of the extent and dosage of neoadjuvant chemoradiation or anastomotic techniques with the lowest leakage rates remains controversial [19, 24].

Most post-SG leaks (>90%) and RYGB leaks occur at the angle of His where the staple-line meets the gastroesophageal junction [25, 26], an area of intense intragastric pressure, thin gastric wall, susceptibility to ischemia owing to the single blood supply to the gastric pouch, as well as relative dysmotility. However, SG leaks may occur anywhere along the length of the sleeve at the staple-line. RYGB leaks may occur also at the gastrojejunal anastomosis, blind loop, jejunojejunal anastomosis, or remnant stomach.

## 3. Endoscopic Armamentarium

**Figure 2:**
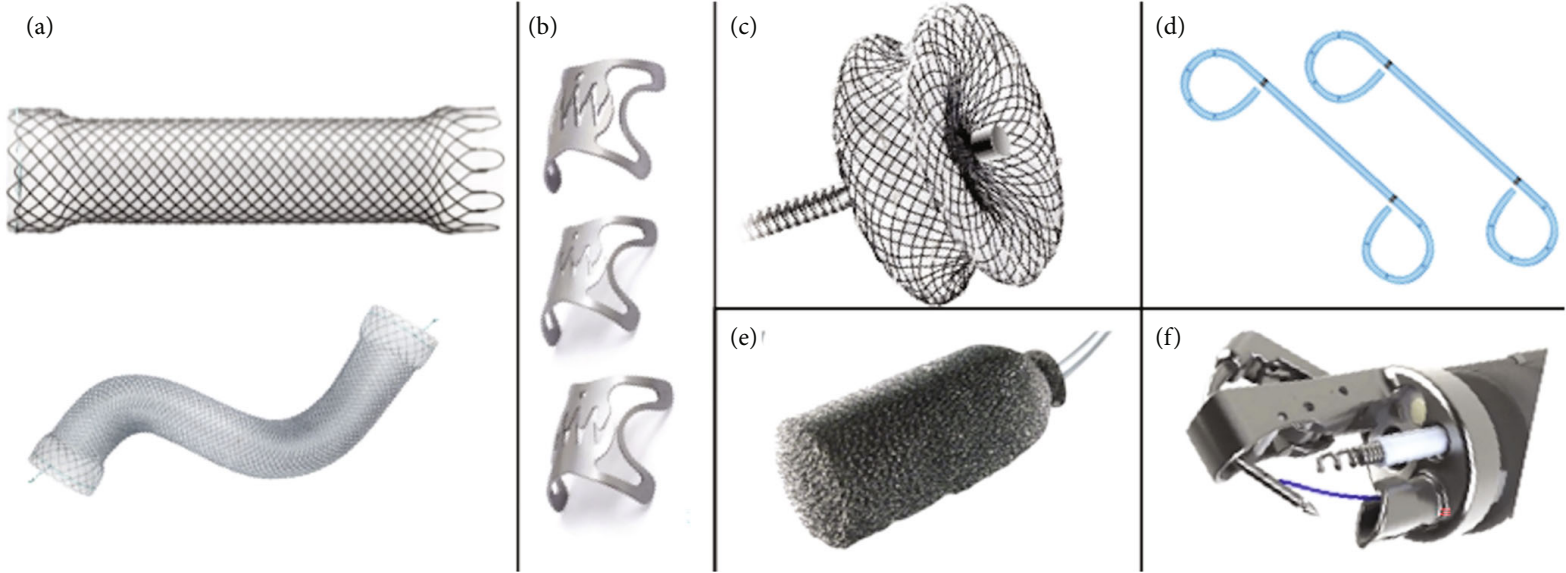
Different examples of endoscopic devices available for treatment of postsurgical leaks. Esophageal self-expandable metal stent and “bariatric stent” (a); different types of over-the-scope clips teeth configurations (b); cardiac septal defect occluder (c); two double plastic pigtail stents (d); polyurethane foam sponge of endoscopic vacuum therapy (e); and endoscopic suture system (f).

Endoscopic techniques (Figure 2) for PSL closure include (Table 2):

1. Close-cover-divert approaches (primary techniques): use of suturing devices, over-the-scope clips (OTSCs), tissue sealants, cardiac septal defect occluder (CSDO), or self-expandable metal stents (SEMSs).
2. Active or passive internal drainage approaches (secondary techniques): endoscopic internal drainage (EID), endoscopic vacuum therapy (EVT), or septotomy with or without pneumatic dilation.

In recent years, leak management has started to fall in the close-cover-divert approach versus the active or passive internal drainage approach.

### 3.1. Stents

**Figure 3:**
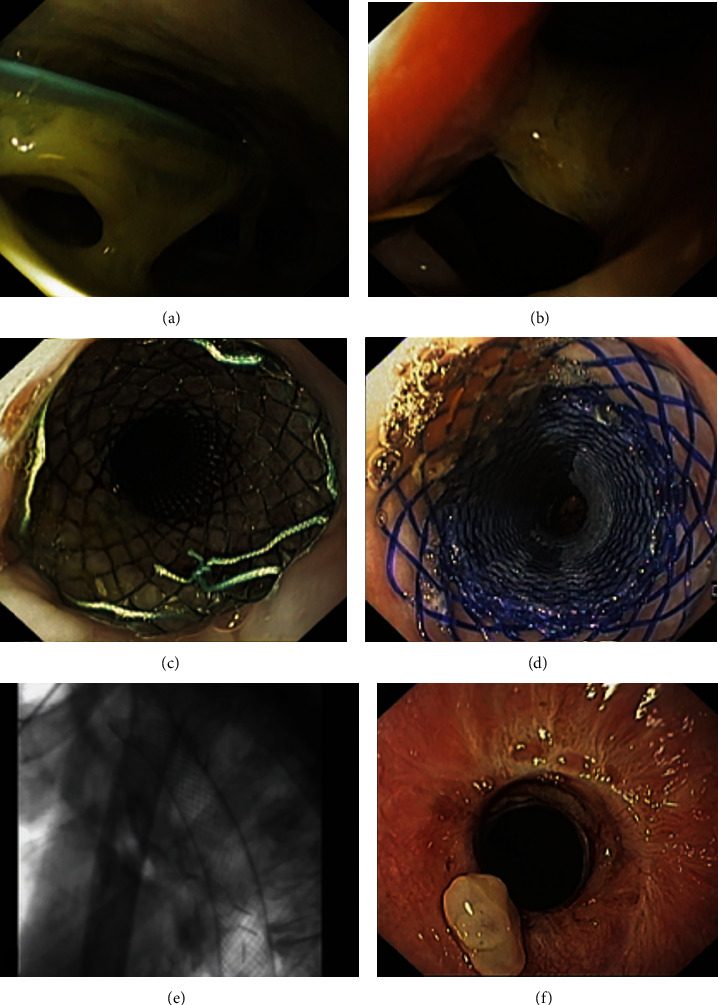
Endoscopic image of a post-total gastrectomy anastomotic leak occupying nearly 50% of the luminal circumference (a). Leak defect and efferent limb with a guidewire in place to guide sent placement (b). Examples of a fully covered self-expandable metal stent (c) and a biodegradable stent (d), which can be used for leak diversion. Fluoroscopic image after SEMS placement (e). Stent-induced stricture at the previous location of the proximal stent flange after stent removal (f).

Endoscopic stents are cylindrical devices used to preserve or re-establish luminal patency [27]. For PSL, the role of a stent is to seal the leak and divert gastrointestinal contents away from the site of leakage, enabling an early resume of enteral feeding [28] (Figure 3). This is an off-label use of these devices. Selection of the ideal stent requires an understanding of stent technology, such as stent's type, dimensions, and degree of foreshortening, as well as location and features of the targeted defect.

Recent esophageal SEMSs are usually made of nitinol, an alloy of nickel and titanium, allowing flexibility for placement at sharp angles [29, 30]. Esophageal SEMS can be partially (PC) or fully covered (FC). The silicon coating completely covering the FC-SEMS is intended to easily remove the stent, but this advantage is overshadowed by the higher migration risk (up to 30%). PC-SEMS may be preferable to FC-SEMS as tissue hyperplasia forms at the uncovered terminal ends of the stent, creating a watertight seal around the stent and decreasing the risk of migration. The major drawback of PC-SEMS is the difficulty of stent removal [31], however, this can be overcome using auxiliary techniques, such as argon plasma coagulation (APC) [32], inversion of the stent by its distal end [33], or the *stent-in-stent* technique [34, 35]. Stent dwell time is highly variable and may range from 2 to 12 weeks [36], even though median time to achieve healing is usually 4–8 weeks [37].

Self-expandable plastic stents (SEPS) consist of a polyester body covered with silicone to prevent tissue ingrowth and polyester braids on the surface to prevent stent migration. Despite SEPS effectiveness in sealing transmural defects, they also have propensity for migration [38] and require mounting on a delivery system before deployment, making the process complicated and time consuming when compared with SEMS, which are ready for use [29]. Thus, SEPS use has largely been replaced by SEMS.

Biodegradable stents (BDSs) are absorbable stents that degrade within 6–24 weeks. Since degradation is accelerated by acid exposure, acid-suppressive therapy may be warranted in certain situations [39]. BDSs negate the inconvenience of stent removal; however, the severity of tissue hyperplasia cannot be accurately predicted [40] and may result in dysphagia and stenosis that requires dilation in approximately 50% of cases [31]. In addition, radial force of BDS is weaker when compared to SEMS [41].

Three systematic reviews comparing the use of PC-SEMS, FC-SEMS, and SEPS in oncologic leaks and perforations [42–44] reported a clinical success of 81–87% without differences among stent types. Despite the non-negligible rate of SEMS-related adverse events (AEs), most of them are usually mild and can be managed conservatively. Nausea, vomiting, and abdominal discomfort are common and usually transient, but severe stent intolerance has been reported, leading to early stent removal. Severe bleeding and perforation may also occur [35, 42–45], but high rate of migration stands as the major drawback. Regarding bariatric leaks, leak closure rates and AEs rates range from 65% to 100% and 14% to 86%, respectively, with migration being the most frequent AE, with rates of 5–67% [42, 45–56]. Recent reports using specifically long and larger designed stents (“bariatric stents”) show similar success rates without significant differences in migration rate when compared to conventional stents [45, 57].

Longer delays until stent placement [58], persistent leakage after initial stent [59], leaks of the proximal esophagus, stents traversing the gastroesophageal junction, defects larger than 6 cm, and distal conduit leaks [60] are associated with higher probability of treatment failure in oncologic leaks. Regarding bariatric leaks, defects larger than 1 cm [61] and longer delays between leak development and stenting also negatively influence endoscopic outcomes [42, 46, 62].

### 3.2. Over-the-Scope Clips

OTSC is a memory-shape nitinol clip, with a “bear claw” configuration and a powerful compression force, loaded onto a transparent cap that is mounted at the tip of the endoscope [63]. They are available on various diameters (OTSC caps of 11, 12, and 14 mm internal diameters) and lengths (3 and 6 mm cap depth), as well as three types of teeth configuration, which include the blunt or atraumatic type (A type), the traumatic type with short pointed teeth (T type), and the traumatic type with long pointed teeth (GC type) [64]. The set-up and deployment of the OTSC are similar to a variceal band ligator, as the cap pulls in the target tissue or defect using vacuum suction. Auxiliary devices, like the tri-prong anchor retraction device (if the tissue is indurated and scarred) or “twin grasper” forceps (to approximate the opposite edges of a pliable gaping defect), may facilitate efficient pulling of the entire defect into the cap [64].

Placement of the OTSC may be challenging due to limited access, restricted mobility, and suboptimal alignment with the target lesion. A misdeployed clip makes subsequent repair very difficult. If misdeployment occurs, OTSC may be removed with high power APC (with the potential for transmural burn injury and delayed perforation), or with a dedicated device (remOVE system, Ovesco, Tübingen, Germany) based on a fast and efficient direct current [65]. Application of ice-cold normal saline on the clip for 1 minute, to lower the mechanical resistance of the nitinol frame prior to its extraction by a standard grasping forceps, has also been reported [66].

Closure of large defect that requires more than one OTSC may not be effective as the concave configuration of these clips results in a gap between two closely placed clips. Another caveat during placement is inadvertent entrapment of the auxiliary devices during deployment, if not fully retracted into the cap. OTSC placement requires care, as surrounding healthy and pliable tissue can easily be suctioned inadvertently into the cap and, if passed unrecognized, result in incomplete luminal closure following clip deployment.

**Figure 4:**
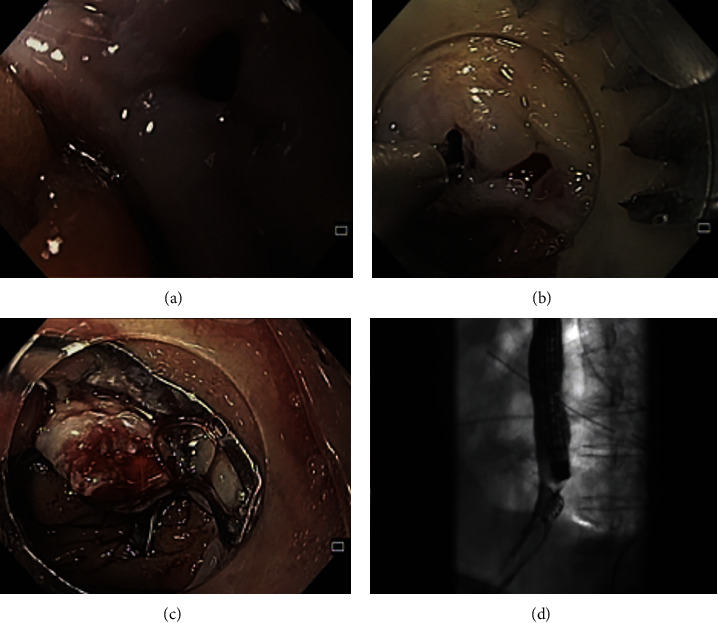
Endoscopic image of a 3 mm leak orifice after total gastrectomy (a), closed with a 12 mm over-the-scope clip (OTSC) after retracting the tissue margins with an anchoring device and suction of the defect into the applicator cap (b, c). Fluoroscopic image showing OTSC correctly placed, without leakage (d).

OTSCs should be used in situations where the tissue margins are still malleable and the entire target defect can be suctioned or retracted into the cap (Figure 4). They are usually reserved for completion closure of large anastomotic leaks that have been reduced by other measures until the defect size is small enough to be amenable for OTSC closure.

Clinical success ranges between 66–73% for oncologic leaks [67, 68] and 67% for post-bariatric leaks [69]. Unfortunately, success rate of post-esophagectomy leaks is below 33%, probably due to the anatomical features of the esophagus (narrow lumen). Clinical success is higher when OTSC is used within 1 week of diagnosis, if applied as primary therapy and if the defect has minimal inflammation or low level of fibrosis [67, 70, 71]. Larger defects (>13 mm) and necrotic or soft margins are associated with increasing failure rates [70].

### 3.3. Endoscopic Suturing

Presently, most experience is limited to the OverStitch device (Apollo Endosurgery, Austin, Texas), which requires a single or double channel therapeutic gastroscope and familiarity with the multistep process associated with activation of the device. The suturing system enables placement of polypropylene or polydioxanone sutures in an interrupted or continuous fashion without the need to remove the endoscope for suture reloading [64]. Accessories, such as the helix device, can be used to anchor and retract tissue into the suturing arm to facilitate suture placement.

The recently developed X-Tack Endoscopic HeliX Tacking System, a Through-the-scope (TTS) suture-based device, allows closure of large, wide, and irregularly shaped defects, without the need for instrument withdrawal from the patient [72]. The tacks are screwed into healthy target tissue adjacent to the defect or stent, followed by approximation of the margins by successive gathering of the tacks with applied suture tension and placement of a final cinch to secure the construct [72, 73].

Available data on endoscopic suture are limited, and results are not satisfactory, since the largest study reported a clinical success of only 27% in leak closure [74].

### 3.4. Tissue Sealants

Tissue compatible glues are either derivative of proteins involved in coagulation or glue such as cyanoacrylate. Fibrin glue, which consists of human fibrinogen and thrombin combined with antifibrinolytic agents, is the most commonly used sealant. It is a tissue-compatible adhesive that mechanically occludes the wall defect and promotes wound healing by inducing cellular response to tissue damage and forming matrix-building strands [75]. Although fibrin glue contains antifibrinolytic agents, accelerated degradation particularly in the setting of gastrointestinal contents or infection remains a concern and, therefore, fibrin glue is considered a poor scaffolding material. Owing to these concerns, recent studies have evaluated infill materials, such as absorbable Vicryl mesh or Surgisis (Biodesign, Cook Medical Inc, Bloomington MA) [76].

Cyanoacrylate, a synthetic glue working as a mechanical sealant, has high adhesive and high antibacterial properties and thus is suitable for application in infectious sites. It is eliminated by hydrolysis after 1–6 months [77]; however, the poor mechanical properties of the film, brittle nature, possible proinflammatory effect as well as the risk of damage of the endoscope because of its rapid polymerization make cyanoacrylate a second-choice method [78].

**Figure 5:**
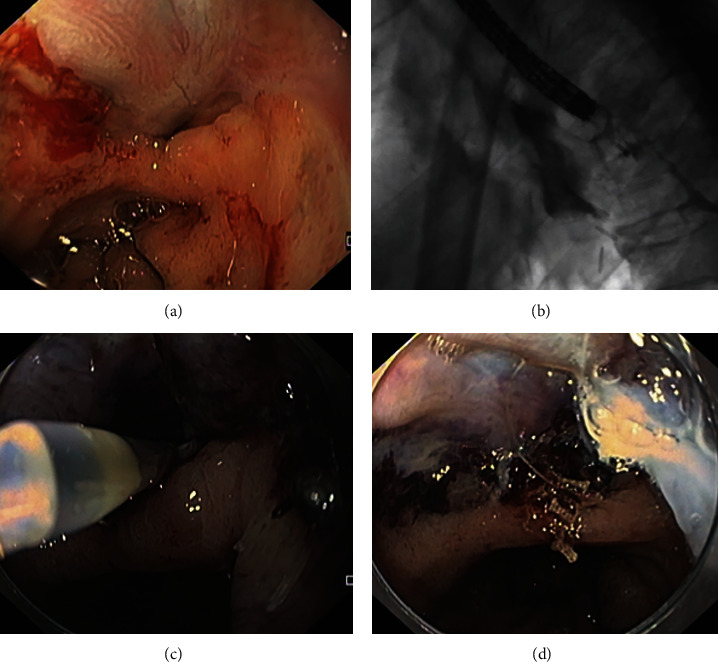
Endoscopic image of a 4 mm anastomotic leak after Ivor-Lewis esophagectomy (a), with associated fistula (b), closed after tissue sealant and vicryl mesh placement (c, d).

Clinical success of glue sealants is highly variable, ranging from 55.7% to 96.8% [77, 79–81]. Glue sealants are frequently used as an adjuvant to other techniques, making difficult to evaluate its efficacy as primary treatment [77, 78]. It might be more suitable for small leaks (<15 mm) or residual small collections after the use of other techniques [78]. Complete leak closure might require multiple sealant applications or the use of vicryl plugs to improve effectiveness (Figure 5) [79].

### 3.5. Cardiac Septal Defect Occluder

The Amplatzer CSDO (St. Jude Medical, Plymouth, MN) is a self-expandable double-disc (“double umbrella”) closure device made of a shape-memory nitinol wire mesh with interlaced polyester, which promotes occlusion and tissue ingrowth [82]. It can easily be recaptured and redeployed for optimal placement. There are two types of CSDO, the atrial septal and the ventricular septal defect closure devices; both are available in different sizes, including disc diameter (from 9 to 54 mm), waist length, and waist diameter (from 4 to 38 mm). To select the adequate CSDO size, estimation of fistula orifice can be made by the ability to pass the gastroscope through the orifice. Whenever possible, waist size should be adjusted to fistula diameter to ensure a tight seal. In addition, a device diameter at least 50% larger than the fistula orifice helps to optimize the seal [83].

The delivery system sheath size ranges from 5 to 12 French (Fr) with a tip angle of 45 and 180 degrees and with a length from 60 to 80 cm, precluding to be used TTS channel of most available gastroscopes. To overcome this limitation, CSDO can be delivered over a guidewire under direct endoscopic visualization with or without fluoroscopy guidance or can be separated from the delivery system and loaded to an adapted endoscopic biliary catheter (7–10 Fr) to enable enough length to be deployed TTS channel. To load the CSDO into the biliary catheter, a pediatric biopsy forceps can be placed down the catheter to grab and back-load the stent [84].

During the deployment, the distal flange is first released into the GI lumen or the fistula tract (if advanced from the skin or the endoscope, respectively) and then, after confirmation of adequate positioning, the proximal flange is released [85].

A systematic review [83] reported a technical success rate of 100% and a clinical success of 77.27%. The largest available study [84] reported a clinical success of 90.7%. Fistula chronicity and previous treatment were associated with increased rates of fistula closure. AEs may occur in up to 23%, mostly migration and, more rarely, fistula enlargement [84].

### 3.6. Endoscopic Vacuum Therapy

**Figure 6:**
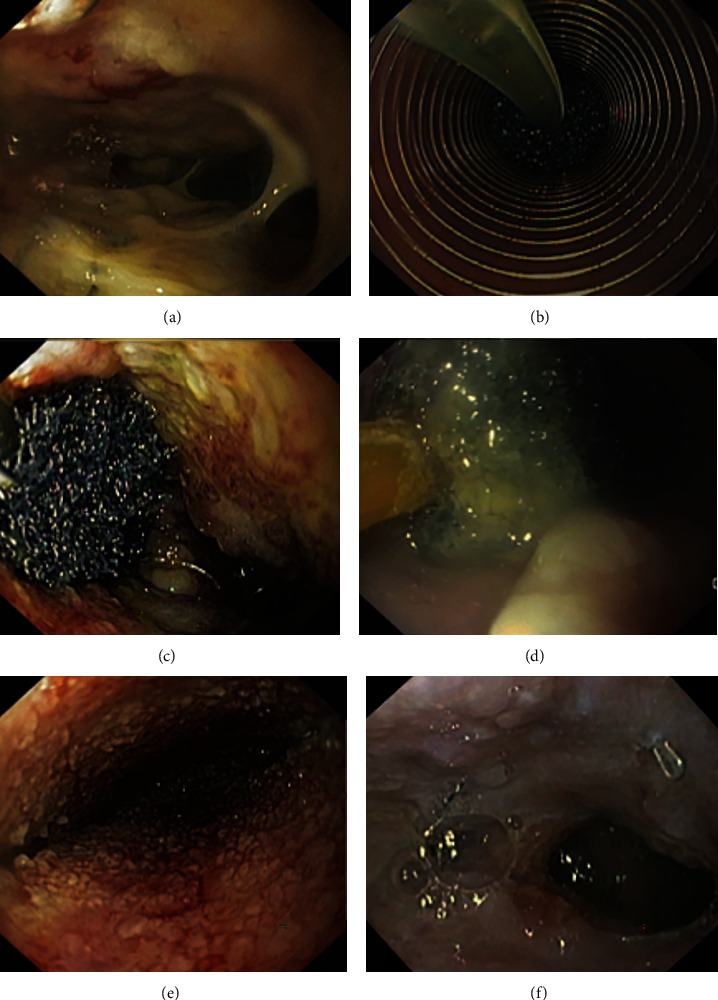
Endoscopic image of a severe anastomotic leak after Ivor-Lewis esophagectomy (a). An overtube (b) was used to assist in the placement of a polyurethane sponge well deep in the mediastinal cavity (intracavitary EVT) (c). EVT sponge during scheduled replacement after 3 days in place (d), with progressive decrease of cavity dimensions and granulation tissue formation (e). Complete closure of the anastomotic defect was achieved (f).

EVT consists in a negative pressure system that promotes wound healing by draining inflammatory exudates and secretions, decreasing bacterial contamination, and promoting neovascularization and granulation tissue with subsequent epithelialization (Figure 6) [19, 86]. In EVT, a polyurethane foam sponge, slightly smaller than the wound's dimensions and geometry (to allow collapse and subsequent closure), is attached at the tip of a polyvinyl chloride suction tube using sutures applied at the proximal and distal ends of the sponge [87–89]. At every endoscopic session, the sponge size should be tailored to the new wound size dimensions.

The two most common techniques used to place the sponge are the back-pack method (dragging the sponge drainage system parallel to the endoscope using an endoscopic forceps) and the overtube method (pushing the sponge down through the tube) [87–91]. If the wound cavity has a narrow opening, it can be endoscopically dilated to facilitate placement of the sponge. However, if the extraluminal cavity is small, the sponge may be placed intraluminally adjacent to the cavity. Negative continuous pressures of 100–125 mm Hg are usually selected [90, 91].

After initial placement of the EVT system, the sponge is changed regularly every 3–4 days for intracavitary sponges (to prevent granulation tissue ingrowth that makes the removal of the sponge difficult) and up to 1-week interval for intraluminal sponges [92], until satisfactory cavity closure is achieved. During this process, the sponge should be changed from its initial intracavitary location to an intraluminal one, and subsequently removed once the cavity has reduced to a radius <1 cm and a depth <2 cm, with formation of a pseudodiverticulum or a rather small opening, which can later be closed using, for example, an OTSC. With the concomitant use of antibiotics and adequate nutritional support through tube feeding, defect closure using the EVT technique can generally be achieved within 15–30 days [93].

Clinical success ranges from 66.7% to 100% [94–96]. Regarding oncologic leaks, clinical success is higher for gastrectomy leaks comparing to esophagectomy leaks (90% vs. 79.5%) [97]. Neoadjuvant treatment, rescue application, and intraluminal location are risk factors for EVT failure [98]. Other limitations associated with EVT should be considered. First, a transnasal tube must remain in situ for at least 3–4 weeks. Second, multiple endoscopic sessions are required. Third, an anatomically difficult to access cavity due to its narrow opening needs endoscopic dilatation (with potential for AEs), whereas a small cavity warrant placement of the sponge intraluminally, which may be less efficient at absorbing secretions and collapsing the cavity [93].

EVT-related AEs (4.1–12%) are usually minor and related to mild bleeding upon sponge exchange, sponge dislodgement, and discomfort or distress from repeated procedures [99]. Stricture formation after EVT, secondary to vigorous formation of granulation tissue, may occur, requiring endoscopic dilation [100]. Rarely, major events like bleeding from sponge erosion into small or major cardiovascular structures, rupture of the descending aorta, or bronchoesophageal fistula formation may occur [90, 95, 101].

### 3.7. Endoscopic Internal Drainage

**Figure 7:**
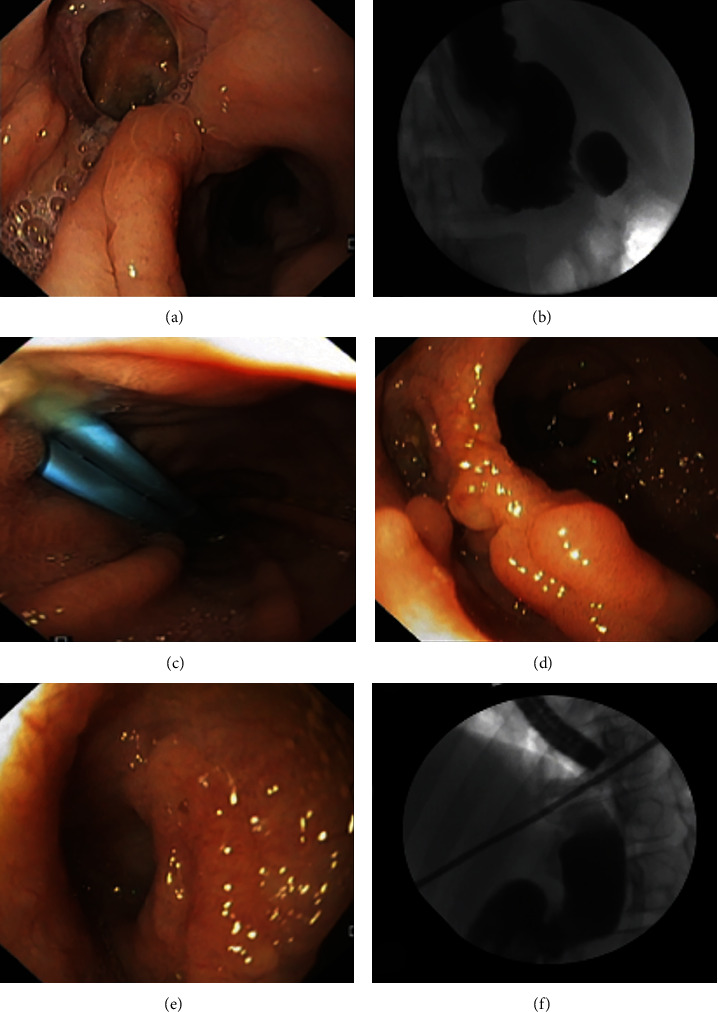
Endoscopic image of a 12 mm anastomotic leak after sleeve gastrectomy (a), with an associated perigastric collection (b). Two plastic double pigtail stents were placed across the leak orifice to internally drain the collection (c). At the end of treatment, a pseudodiverticulum formation in the previous anastomotic leak location could be seen (d, e), and successful closure of the defect was achieved (f).

The rationale of EID with deployment of one or more pigtail plastic stents (or nasocystic catheters in cases of large collections requiring lavage to eliminate pus and debris [16]) across the leak orifice is to internally drain fluid collections, leading to progressive reduction in leak size until it eventually becomes a virtual cavity (Figure 7) [102]. Meanwhile, a foreign body reaction in the edges of the leak is triggered by the pigtail stents, promoting re-epithelialization and leak closure, resulting in an all-in-one procedure without the need of further treatment. A residual small cavity like a pseudodiverticulum is common at the end of the process without any clinical repercussion [103]. In addition to stenting, debridement (endoscopic necrosectomy) may also be needed in cases of infected collections containing necrotic tissue [104–106]. Downstream stenosis in the gastric lumen should be treated if present, to reduce the intragastric procedure.

The appropriate time interval for stent exchange or oral diet resumption remains to be defined. While stent exchange may be performed on a regular basis (i.e., every 2–6 weeks, until healing is achieved), to avoid stent obstruction, allow necrosectomy, and stimulate tissue granulation [103], others remove the stents 4 months after complete clinical resolution [16], even though in most patients successfully treated, stents often migrate spontaneously. Oral diet is usually started in the first 24–48 hours after confirming clinical improvement with EID [16] or following confirmation of collection reduction in CT scan [103].

Clinical success of EID ranges from 78–86% with a median time to leak closure up to 115 days [16, 102, 103]. Discomfort, ulceration, dysphagia, and splenic hematoma are rare EID-related AEs [16]. When combined with surgery cleansing, EID allows early removal of surgical drainage preventing chronic fistula tract formation [107]. Longer delays between diagnosis and treatment, larger leaks, sepsis, presence of gastrobronchial fistula, and previous OTSC deployment are risk factors for treatment failure [14].

### 3.8. Endoscopic Septotomy

This procedure derives from the endoscopic treatment for Zenker diverticulum. The principle behind this technique relates to higher intraluminal pressure within the sleeve compared with the perigastric cavity, promoting flow of contents through the leak. Endoscopic septotomy aims to equalize these pressures by cutting the “septum” between the perigastric cavity and the gastric lumen, using APC or a needle knife, allowing internal drainage of the leak, and deviation of oral intake. The cut should not exceed the bottom of the perigastric cavity. If a downstream stenosis in the gastric lumen is present, it should be treated as well, similarly to EID. Multiple endoscopic procedures may be required with more pseudo-septum being incised each time to achieve successful healing [22].

Endoscopic septotomy may be used as first-line or salvage therapy with clinical success ranging from 70% to 85% [11, 107–109]. Bleeding and perforation should be taken as potential AEs [110].

## 4. Discussion

Therapeutic endoscopy plays a major role in the management of PSL, offering an effective treatment alternative to repeat surgery [110]. Despite this, there is wide variation in the management of these patients, even among experts in the field, particularly concerning difficult-to-treat patients. Proper selection of patients is critical for favourable outcomes, and the approach to UGI PSL should always be tailored to the single patient. So, it requires a personalized and multidisciplinary approach, comprising a close collaboration between interventional endoscopist, radiologist, and surgeon, allowing PSL management with high clinical success rate and low rate of morbidity and mortality [111, 112].

A single therapy, or a combination of different techniques, can be used for PSL treatment. In fact, most patients may benefit from a multimodal approach. However, leak resolution seems to reach a plateau between third and fourth endoscopic techniques used [113]. Despite no definitive consensus on the definition of endoscopic failure, persistent inflammation with clinical sepsis, and impossibility to resume oral feeding should be considered (Table 3) [114]. Inability to close the leak with time, especially after 4 months of treatment, should also prompt consideration of therapeutic alternatives, namely surgery [114].

It is important to highlight that surgery still has a key role in addressing PSL, both at the initial stages (allowing irrigation and drainage of intrathoracic or intra-abdominal collections) and at later stages if endoscopic treatment is not successful. Outcomes of salvage surgical procedures may be exaggerated due to selection bias, as patients are generally sicker or have failed multiple previous therapies [115].

**Figure 8:**
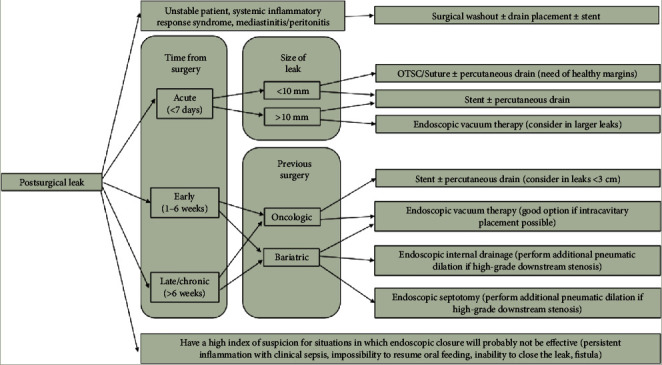
Endoscopic algorithm for management of postsurgical leaks. Combined treatment with simultaneous or sequential use of several endoscopic methods should always be considered.

To summarize, when approaching PSL, the following principles should be considered (Figure 8):

- Referral of leaks for endoscopic treatment should be as soon as possible.
- In patients whose condition is unstable, with acute leaks and systemic inflammatory response syndrome or mediastinitis/peritonitis, surgical washout with or without drain placement is mandatory and should not be delayed. Concurrent endoscopic management with stent placement may also be effective in this setting, before the formation of an organized collection.
- Combined treatment with simultaneous or sequential use of several endoscopic methods may be optimal.
- Symptomatic and small (<10 mm) acute leaks, with healthy defect margins, may be considered for stenting, OTSC, or suture. Stenting may be a better option for intrathoracic leaks, while OTSC and suture may be more suitable for intra-abdominal leaks.
- For acute lesions with nonviable margins or size >10–15 mm, stenting or EVT can be considered. EVT might be a superior tool for the management of cervical leaks, larger leaks (>3 cm), and chronic leaks.
- EID may be considered for the management of subacute or chronic post-bariatric leaks with an organized walled-off collection. If this fails, EVT should be considered.
- Endoscopic septotomy may be performed in late or chronic sleeve leaks with organized walled-off collections, especially if failure of other techniques.
- In patients with post-SG leaks with high-grade downstream stenosis, additional pneumatic dilation with a balloon is required.
- In the setting of associated collections, if closure techniques are used, external drainage is required. EID and EVT allow early removal of external drainage preventing chronic fistula tract formation.
- OTSCs and tissue sealants may be considered for closure of residual small collections after the use of other techniques.
- Have a high index of suspicion for situations in which endoscopic closure will probably not be effective. These situations include persistent inflammation with clinical sepsis, impossibility to resume oral feeding, inability to close the leak (especially after 4 months of treatment), and formation of enterocutaneous or enteropleural fistulas.

## Figures and Tables

**Table 1:** Clinical success rates of different endoscopic techniques in the management of upper gastrointestinal postsurgical leaks.

Endoscopic technique	Clinical success rate	
	Oncologic leaks	Bariatric leaks
Stents	81–87% [41–43]	65–100% [41, 44–55]
OTSC	66–73% [65, 66]	67% [67]
Suture	27% [72]	
Tissue sealants	56–97%∗ [75, 77–79]	
CSDO	77.3% [81]	
EVT	67–100% [92–94]	
EID	76–86% [16, 100, 101]	
Endoscopic septotomy	70–85% [11, 105–107]	

∗Frequently used as an adjuvant of other techniques.

OTSC: over-the-scope clip; CSDO: cardiac septal defect occluder; EVT: endoscopic vacuum therapy; EID: endoscopic internal drainage.

**Table 2:** Summary of endoscopic treatment devices for management of upper gastrointestinal postsurgical leaks.

Type	Device	Advantages	Disadvantages
Primary closure techniques	Over-the-scope clips	Useful for small leaks [68, 76]	Frequent need of combined techniques [75, 76]
	Tissue sealants	Extensively available in most centers [62]	Less effective for larger and chronic leaks [68]
		Ease of use [62]	
	Endoscopic suture	Useful for small leaks [70]	Less effective for larger and chronic leaks [72]
			Not widely available [72]
			Requires experienced endoscopist [72]
			Poor clinical success [72]
	Cardiac septal occluded	Useful for leaks with associated fistulas [81]	Less effective for larger leaks [81]
			Requires experienced endoscopist [82]
	Self-expandable metal stent	Early enteral nutrition [27]	Frequent but transitory symptoms after stent placement (nausea, vomiting, and/or retrosternal discomfort) [34]
		Widely available in most centers	Multiple endoscopic sessions (larger leaks) [60]
		Allow simultaneous dilation if concomitant stricture is present [28, 29]	Stent migration risk despite fixation [37]
			No consensus about best stent type [41–43]
Secondary closure techniques	Endoscopic vacuum therapy	Combines drainage and sealing [18]	Transnasal tube in situ for at least 3–4 weeks [90]
		No need for percutaneous drain [84]	Multiple endoscopic procedures every 3–4 days (sponge exchange) [90]
		Possibility of closure of larger and chronic defects [85]	Late enteral nutrition (total parenteral nutrition or jejunostomy is needed) [91]
	Endoscopic internal drainage	No need for percutaneous drain [100]	Long period till leak closure [100]
		Early oral feeding [16]	Complementary techniques may be needed (necrosectomy/endoscopic ultrasound guided drainage for complex collections) [102–104]
		Early hospital discharge [16]	
	Endoscopic septostomy	No need for percutaneous drain [21]	Multiple endoscopic procedures may be required [21]
		Option in chronic refractory leaks [105]	Risk for perforation and/or bleeding [108]

**Table 3:** Risk factors associated with endoscopic treatment failure.

Endoscopic technique	Risk factors for treatment failure
Stents	Delay until stent placement [57]
	Persistent leakage after first stent placement [58]
	Proximal esophagus leak [59]
	Larger defect (>60 mm—oncologic; >10 mm—bariatric) [59, 60]
	Stent crossing the gastroesophageal junction [59]
	Distal conduit leak [59]
OTSC	Larger defect (>13 mm) [68]
	Necrotic or soft margins [68]
	Post-esophagectomy leak [65, 66]
EVT	Neoadjuvant chemotherapy [96]
	Rescue application [96]
	Intraluminal sponge placement [96]
EID	Delay until treatment [14]
	Larger leak [14]
	Sepsis [14]
	Gastrobronchial fistula [14]
	Previous OTSC use [14]
Endoscopic septotomy	Persistent stricture below the leak [21]

OTSC: over-the-scope clip; EVT: endoscopic vacuum therapy; EID: endoscopic internal drainage.

## Data Availability

Data supporting this research article are available from the corresponding author or first author on reasonable request.
